# The Analgesic and Side Effects of Tramadol Hydrochloride Immediate-Release and Extended-Release Tablets in Patients With Chronic Low Back Pain

**DOI:** 10.7759/cureus.72124

**Published:** 2024-10-22

**Authors:** Kazuhide Inage, Takeshi Sainoh, Kohei Okuyama, Masaomi Yamashita, Shuhei Ohyama, Kazuki Fujimoto, Hiroto Chikubu, Koki Abe, Seiji Ohtori, Sumihisa Orita

**Affiliations:** 1 Orthopaedic Surgery, Graduate School of Medicine, Chiba University, Chiba, JPN; 2 Orthopaedic Surgery, Sainou Hospital, Toyama, JPN; 3 Orthopaedic Surgery, Funabashi Central Hospital, Funabashi, JPN; 4 Orthopaedic Surgery, Kohnodai Hospital, National Center for Global Health and Medicine, Chiba, JPN

**Keywords:** analgesic, low back pain, patients, side effect, tramadol

## Abstract

Overview of literature: There are no established treatments for low back pain. Conventional treatments include physiotherapy and pharmacological treatments, such as non-steroidal anti-inflammatory drugs (NSAIDs). Recently, analgesics with mechanisms of action different from those of NSAIDs have emerged. Tramadol formulations have especially attracted attention for being effective among patients in whom NSAIDs are not entirely effective. However, they are associated with side effects that can disrupt treatment. Tramadol hydrochloride immediate-release and extended-release tablets have been recently launched. Herein, we report our experience with these formulations in chronic low back pain patients.

Purpose: To investigate the incidence of side effects and the analgesic effect of tramadol hydrochloride immediate-release and extended-release tablets in patients with chronic low back pain.

Methods: This was a multicenter retrospective observational study. The study included patients prescribed tramadol hydrochloride immediate-release and extended-release tablets for chronic low back pain at 13 facilities in Japan between January 2021 and December 2023. The primary outcome was the incidence of side effects observed during the study period. The secondary outcomes were changes in the visual analog scale (VAS) score.

Results: The incidence of side effects was 37.7% (40/106 cases), which included constipation, nausea, drowsiness, and dizziness in 23 (21.7%), 13 (12.3%), 4 (3.8%), and 2 (1.9%) cases, respectively. The rate of treatment discontinuation owing to side effects was relatively low (16/106 cases, 15.1%). Furthermore, after four weeks of treatment, we found a significant reduction in the VAS scores for low back pain from baseline.

Conclusion: This study shows the incidence of side effects following the administration of tramadol hydrochloride immediate-release and extended-release tablets. Regarding the breakdown of side effects, constipation, nausea, drowsiness, and dizziness were noted. Regarding pain evaluation, we found a significant reduction in the VAS scores for low back pain from baseline after four weeks of treatment, suggesting that tramadol hydrochloride immediate-release and extended-release tablets may provide excellent analgesic effects owing to their pharmacokinetic properties that ensure stable tramadol concentrations in the bloodstream.

## Introduction

The lifetime prevalence of low back pain is estimated to be 80%, accounting for the majority of musculoskeletal disorders [1]. Furthermore, only approximately 20% of cases can be definitively diagnosed through imaging and other diagnostic methods because non-organic diseases are more prevalent than organic diseases, often making treatment challenging [2]. Currently, there are no established fundamental treatments for non-specific low back pain, and socioeconomic losses owing to chronic low back pain are immeasurable [3]. Additionally, while relief from acute low back pain may be achieved quickly, chronic low back pain tends to be more treatment-resistant [4,5].

Conservative treatments for chronic low back pain primarily consist of physiotherapy, as well as pharmacological treatments, such as the oral administration of non-steroidal anti-inflammatory drugs (NSAIDs). In recent years, analgesics with mechanisms of action different from those of NSAIDs have emerged, marking a significant turning point in the pharmacotherapy of chronic low back pain. Particularly, tramadol formulations, which are indicated for non-cancerous pain, have attracted attention. Tramadol is reportedly effective in the management of chronic pain among patients in whom sufficient pain control was not achieved using NSAIDs, and its prescription has been increasing in the field of orthopedics [6,7]. However, tramadol formulations are also associated with a high frequency of opioid-specific side effects, such as nausea and constipation, which can lead to discontinuation of the medication. Additionally, in Western countries where tramadol is frequently prescribed, drug abuse is a prevalent issue, necessitating caution.

New tramadol formulations, such as tramadol hydrochloride immediate-release and extended-release tablets, have been launched in this context. Tramadol hydrochloride, the first formulation of its kind to be administered twice daily in Japan, is available as a tablet with a dual structure comprising an immediate-release layer on top, an extended-release layer on the bottom, and a film coating. The action of this drug is characterized by a shorter time to reach the maximum blood concentration (tmax) compared to existing tramadol formulations and a tendency for a longer half-life (t1/2) than that of immediate-release formulations. These pharmacokinetic properties suggest that this formulation can achieve maximum blood concentration (Cmax) more quickly and maintain blood concentration levels longer than immediate-release formulations [8]. We aimed to investigate the incidence of side effects and the analgesic efficacy of tramadol hydrochloride immediate-release and extended-release tablets in patients with chronic low back pain.

## Materials and methods

This was a multicenter retrospective observational study. This multicenter retrospective observational study was conducted at 13 facilities in Japan. The participants were patients prescribed tramadol hydrochloride immediate-release and extended-release tablets (Nippon Zoki Pharmaceutical Company, Tokyo, Japan) for chronic low back pain between January 2021 and December 2023 at the participating facilities. Chronic low back pain was defined as a condition in which the pain persisted for more than three months, with the diagnosis made by orthopedic specialists in all cases. The following individuals were included: Patients who were started on an initial dose of 50 mg twice per day, in accordance with the package insert (with dose adjustments and tapering left to the discretion of the attending physician); Patients for whom other painkillers were not added or for whom the dose was not increased after the initial dose (continuation was allowed); Patients who underwent side effect monitoring at 2, 4, 8, and 12 weeks after administration; Patients who underwent pain evaluations (visual analog scale (VAS)) before and at 2, 4, 8, and 12 weeks after drug administration and excluded: patients receiving other conservative treatments.

The primary outcome was the incidence of side effects observed during the study period. The secondary outcome was the change in the VAS scores for low back pain.

Ethics statement

Our institutional review board approved the study, and patient consent was obtained via the opt-out method.

Statistical analysis

Independent t-tests were used to statistically analyze the changes in the VAS scores. The level of significance was set at p<0.05.

## Results

The patients’ demographic characteristics are shown in Table 1. Overall, 106 patients (average age: 65.7±16.1 years; sex: 62 men (58.5%) and 44 women (41.5%)) were included. The underlying conditions were lumbar spinal canal stenosis, degenerative low back pain, lumbar spondylolisthesis, muscular low back pain, and discogenic low back pain in 44 (41.5%), 22 (20.8%), 17 (16.0%), 15 (14.2%), and eight (7.5%) cases, respectively. The duration of low back pain was 26.4±23.4 weeks. Prior medication, with some overlap, included NSAIDs, acetaminophen, duloxetine, and neurotropin (a non-protein extract of inflamed rabbit skin inoculated with vaccinia virus) in 59 (55.7%), 17 (16.0%), 13 (12.3%), and 10 (9.4%) cases, respectively. Additionally, 17 (13.5%) cases had no prior medication use. Concomitant medications (with overlap, continued from before the initiation of the trial) included NSAIDs, acetaminophen, duloxetine, and neurotropin in 32 (30.2%), 15 (14.2%), 7 (6.6%), and 6 (5.7 %) cases, respectively; there were 49 (46.2%) cases with no concomitant medication use. The medications used for side effect management included domperidone, metoclopramide, prochlorperazine maleate (for nausea), naldemedine tosylate, and magnesium oxide (for constipation) in 54 (50.9%), 10 (9.4%), 7 (6.6%), 19 (17.9%), and 26 (24.5%)cases, respectively. The incidence of side effects is presented in Table 2.

**Table 1:** Patient background in the present study

n=106		
Age (y/o)	65.7±16.1	
Sex (cases)	Male	Female
	62 (58.5%)	44 (41.5%)
The causative disorders (cases)	Lumbar spinal stenosis	44 (41.5%)
	Spondylosis deformans	22 (20.8%)
	Lumbar spondylolisthesis	17 (16.0%)
	Muscular low back pain	15 (14.2%)
	Discogenic low back pain	8 (7.5%)
The duration of lower limb pain (weeks)	26.4±23.4	
Drugs used for premedication (cases)	NSAIDs	59 (55.7%)
	Acetaminophen	17 (16.0%)
	Duloxetine	13 (12.3%)
	A non-protein extract of inflamed rabbit skin inoculated with vaccinia virus	10 (9.4%)
	No premedication	17 (13.5%)
The concomitant drugs (cases) ※Continuous-use drugs before the test	NSAIDs	32 (30.2%)
	Acetaminophen	15 (14.2%)
	Duloxetine	7 (6.6%)
	A non-protein extract of inflamed rabbit skin inoculated with vaccinia virus	6 (5.7%)
	No continuously used drug	49 (46.2%)

**Table 2:** Occurrence of adverse drug reactions

Adverse drug reactions	40/106 cases (37.7%)
Constipation	23 cases (21.7%)
Nausea	13 cases (12.3%)
Drowsiness	4 cases (3.8%)
Dizziness	2 cases (1.9%)
In 16 out of 106 cases (15.1%), the drug was discontinued because of adverse drug reactions.	

Side effects were observed in 40 of the 106 patients (37.7%) and included constipation, nausea, drowsiness, and dizziness in 23 (21.7%), 13 (12.3%), 4 (3.8%), and 2 (1.9%) cases, respectively. The number of patients who discontinued treatment due to side effects was 16 out of 106 (15.1%), with nausea accounting for 11 cases (10.4%) and constipation for five (4.7%). The timing of side effect occurrence (n = 40) was two weeks (immediately after treatment initiation), 4 and 8 weeks in 32 (80.0%), 6 (15.0%), and 2 (5.0%) cases, respectively.

**Figure 1:**
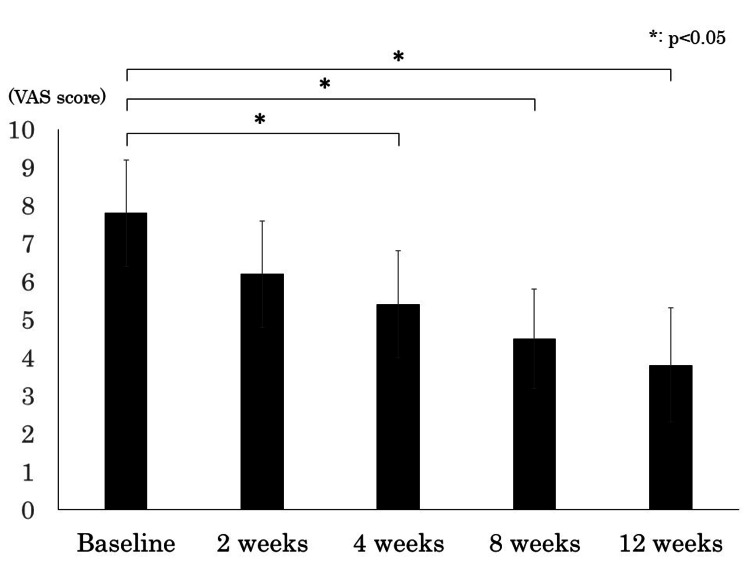
Temporal change of VAS for low back pain VAS: Visual analog scale

For the secondary outcome, pain evaluation (VAS) was performed on 90 patients (84.9%), excluding 16 (15.1%) who discontinued treatment due to side effects. The results showed a significant decrease in the VAS scores for low back pain over time (mean±SD) from 7.8±1.4 points at baseline to 6.2±1.4, 5.4±1.4, 4.5±1.3, and 3.8±1.5 points at 2, 4, 8, and 12 weeks after drug administration, respectively, with a significant reduction observed from four weeks after drug administration onward (p <0.01) (Figure 1).

When comparing the change in VAS scores between the group with other analgesics (57 cases) and without (49 cases), no significant difference was observed in the reduction of VAS scores at 4 and 8 weeks. In the group with side effects, the majority of cases discontinued treatment in the early stages, making it difficult to evaluate VAS scores over time.

## Discussion

In this study, the incidence of side effects following the administration of tramadol hydrochloride immediate-release and extended-release tablets was 37.7% (40/106 cases). Previous clinical trials for other conditions have reported a higher frequency of side effects, with 80.6% in knee osteoarthritis and 78.7% in post-herpetic neuralgia [9,10]. The disparity in results may be attributed to the rigorous protocols of phase III trials conducted in Japan, which included strict management of visit intervals and examination schedules, leading to comprehensive recording of even minor side effects. In contrast, our study, conducted in a real-world clinical setting, may not have captured all side effects as thoroughly. Nonetheless, we believe that our study provides a valuable reflection of the side effects that clinicians actually observe, offering significant clinical insights [11].

Regarding the breakdown of side effects, constipation (21.7%), nausea (12.3%), drowsiness (3.8%), and dizziness (1.9%) were noted. In comparison, the aforementioned clinical trials reported constipation (40.7% and 43.8%), nausea (44.4% and 34.9%), drowsiness (21.4% and 18.5%), and dizziness (8.5% and 11.6%) in patients with osteoarthritis and post-herpetic neuralgia, respectively [9,10]. Although there were differences in the frequencies, the composition of the side effects was similar, highlighting that constipation, nausea, drowsiness, and dizziness were the main side effects, which were also evident in our clinical setting [12].

Interestingly, the rate of treatment discontinuation owing to side effects was relatively low (16/106 cases, 15.1%), suggesting that severe side effects leading to treatment discontinuation are rare or that side effects do not persist for a long time. Nausea was the most common reason for treatment discontinuation, indicating its significant impact on treatment cessation compared with other side effects. This underscores the importance of nausea prevention, such as the appropriate use of antiemetics, as a key factor in continuing medication [13].

Regarding pain evaluation, we found a significant reduction in the VAS scores for low back pain from baseline after four weeks of treatment, suggesting that tramadol hydrochloride immediate-release and extended-release tablets may provide excellent analgesic effects owing to their pharmacokinetic properties that ensure stable tramadol concentrations in the bloodstream. Clinical reports on the analgesic effects of this formulation in conditions such as post-herpetic neuralgia and in patients with chronic pain who did not achieve sufficient relief from using adjunctive analgesics or oral non-opioid analgesics support our findings, showing its superiority to placebo and a sustained reduction in pain scores over time [10,14]. Although these reports differ in terms of target populations, dosages, and treatment durations, they corroborate the potential of immediate- and extended-release tramadol hydrochloride tablets for effective pain management, as indicated in our study. Furthermore, a previous study suggested that combining NSAIDs with low-dose tramadol could reduce adverse events and prevent the transition from acute to chronic low back pain, thus proposing a potentially useful treatment option for patients in whom chronic low back pain is inadequately managed by NSAIDs alone [15,16].

The limitations of our study include its retrospective design, the absence of a control group, a small sample size, and the lack of consideration for the effects of prior medications or dosage variations. Future research should include prospective studies with control groups and larger sample sizes to more thoroughly explore dosage effects. This study was limited to a 12-week observation period without long-term follow-up. As chronic low back pain often requires extended management, future studies should consider longer follow-up periods to assess this treatment's sustained efficacy and safety. Furthermore, the presence of patients using medications for side effect management alongside those not using them was a limitation in this study.

## Conclusions

Our study demonstrated that the administration of tramadol hydrochloride immediate-release and extended-release tablets was associated with a lower incidence of side effects compared to previous clinical trials for knee osteoarthritis and postherpetic neuralgia using the same medication. The most commonly observed side effects were constipation and nausea, followed by drowsiness and dizziness in decreasing order of frequency. While differences in study design and target conditions may have influenced these findings, the results suggest favorable tolerability of these formulations in patients with chronic low back pain. Pain evaluation revealed a significant reduction in VAS scores from baseline after several weeks of treatment, indicating that tramadol hydrochloride immediate-release and extended-release tablets provide effective pain relief, likely due to their pharmacokinetic properties that maintain stable blood concentrations. These findings support this formulation's potential utility in managing chronic low back pain.
